# The Sympathetic Nervous System in Hypertension: Roadmap Update of a Long Journey

**DOI:** 10.1093/ajh/hpab124

**Published:** 2021-08-06

**Authors:** Guido Grassi

**Affiliations:** Clinica Medica, Department of Medicine and Surgery, University of Milano-Bicocca, Milan, Italy

**Keywords:** blood pressure, heart rate, hypertension, iron load, meta-analysis, microneurography, renal denervation, residual risk, sympathetic nervous system

## Abstract

The present paper will provide an update on the role of sympathetic neural factors in the development and progression of essential hypertension by reviewing data collected in the past 10 years. This will be done by discussing the results of the published studies in which sympathetic neural function in essential hypertension and related disease has been investigated via sophisticated and highly sensitive techniques, such as microneurographic recording of sympathetic nerve traffic and regional norepinephrine spillover. First, the relevance of the pathophysiological background of the neurogenic alterations will be discussed. It will be then examined the behavior of the sympathetic neural function in specific clinical phenotypes, such as resistant hypertension, pseudoresistant hypertension, and hypertensive states displaying elevated resting heart values. This will be followed by a discussion of the main results of the meta-analytic studies examining the behavior of sympathetic nerve traffic in essential hypertension, obesity, metabolic syndrome, and chronic renal failure. The sympathetic effects of renal denervation and carotid baroreceptor stimulation as well as the possible involvement of sympathetic neural factors in the determination of the so-called “residual risk” of the treated hypertensive patients will be finally discussed.

Since the publication in 2010 in the *American Journal of Hypertension* of our state-of-the-art paper on the sympathetic nervous system (SNS) in hypertension,^1^ several studies have been published allowing to considerably expand our knowledge in the field and to strengthen some new concepts that 11 years ago were making their initial steps in the world of the SNS in hypertension.

The goal of this paper is to provide a critical overview of the new findings, new concepts, and future directions of the research targeted on SNS in hypertension. The roadmap of the journey will include the following stages. First, we will provide a brief summary on what we regard as well established notions on SNS in hypertension. We will then examine the evolution of the new concepts on the pathophysiology of the SNS activation in the high blood pressure state, highlighting the relevance of the immune system and the inflammatory process in the development of the adrenergic overdrive, but also discussing the potential role of genetic and other factors which directly or indirectly may exert sympathoexcitatory effects. A further step will be to describe the main features of the SNS in resistant hypertension, a clinical condition which became in the past years an area of intense investigation. During the last decade, a consistent amount of interest has been focused on heart rate as potential sympathetic marker, given the evidence that the latest European guidelines on hypertension have recognized the importance of elevated heart rate values as an independent cardiovascular risk factor. Another important step of the long journey will be represented by the results of major meta-analyses recently published aimed at providing conclusive information on how hypertension and other comorbidities may affect the SNS. The last stage of the journey will be devoted to (i) the analysis of the data obtained in the past decade on the involvement of the SNS in the blood pressure effects of bilateral renal nerves ablation (as well as of other invasive approaches) and (ii) the possibility that the so-called “residual risk” displayed by treated hypertensive patient may be at least in part linked to the behavior of the sympathetic responses to antihypertensive drugs.

## LOOKING BACK: A SHORT SUMMARY OF PREVIOUS INFORMATION

**Figure 1.**
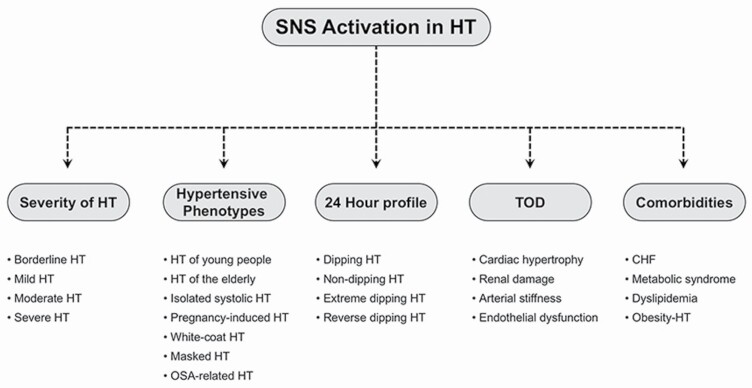
Scheme illustrating the various hypertensive phenotypes characterized by sympathetic activation. Target organ damage (TOD) and comorbidities associated with the adrenergic overdrive are also shown. Abbreviations: CHF, congestive heart failure; HT, hypertension; SNS, sympathetic nervous system.

As shown in Figure 1, a decade ago the information related to the role of SNS in hypertension were derived from the following evidence. First, SNS is activated in essential hypertension, the magnitude of the activation becoming progressively greater from the borderline, mild, moderate to severe hypertensive state.^2^ Second, different clinical phenotypes of hypertension (borderline hypertension, hyperkinetic hypertensive state of the young, middle-age hypertension, systolic hypertension of the elderly, white-coat hypertension, masked hypertension, and obstructive sleep apnea-related hypertension) display as a common feature the SNS activation.^3^ Third, one of the major drivers of the adrenergic overdrive is the 24-hour blood pressure load, which in several clinical conditions appears to be related to the sympathetic function more closely than clinic blood pressure.^3^ Fourth, the detection of associated target organ damage (particularly cardiac and renal) is accompanied by a further sustained increase in the sympathetic cardiovascular drive.^3^ Fifth, not only organ damage but also associated clinical conditions, such as obesity, obstructive sleep apnea, metabolic syndrome, congestive heart failure, and renal failure, may further potentiate the already elevated adrenergic cardiovascular drive detected in uncomplicated essential hypertension.^3^ Some other well-defined aspects of the neuroadrenergic activation are worthy of mention. These include the evidence that different organs (particularly the brain, the heart, and the kidneys) share the hyperadrenergic state, as elegantly documented by the regional norepinephrine spillover technique.^4^ In addition, a number of humoral and reflex mechanisms contribute at the pathophysiology of the neurogenic alterations, which thus appear to have as background a multifactorial nature.^3,4^ Finally, conclusive evidence has been provided that the SNS alterations are not irreversible, but they can be modulated by pharmacological and nonpharmacological interventions currently employed in the therapeutic approach to the hypertensive state.^3,5^

## SNS ACTIVATION: NEW PATHOPHYSIOLOGICAL INSIGHT

The last decade has seen the development of new concepts in the pathophysiological background leading to the neuroadrenergic overdrive in hypertension. These in particular include 3 areas.

The first one refers to the role of immunologic and inflammatory factors, whose importance in the development of the neuroadrenergic activation received in recent years growing experimental support. The current knowledge on this participation can be summarized as follows. An increased number of studies have shown that immune mechanisms play a primary role in the pathophysiology of essential hypertension by contributing via the participation of macrophages and immune competent cells at the inflammatory state which characterizes not only hypertension but the atherosclerotic vascular process and congestive heart failure.^6,7^ Evidence has been accumulated, particularly in essential hypertension, that the relationships between the immune system and SNS are bidirectionally interactive.^8^ Concerning the role of the SNS as triggering mechanism of the interaction, demonstration has been provided that (i) an increase in sympathetic activity elicits T-lymphocytes activation and vascular inflammation,^9^ (ii) significant correlations have been found between circulating plasma norepinephrine and interleukin-6 produced by T-lymphocytes as well as tumor necrosis factor-alpha produced by macrophages and monocytes,^10^ and (iii) chronic SNS activation desensitizes lymphocyte β _2_-adrenoceptors, thereby altering the immune function.^10^ On the other hand, inflammation and T-lymphocyte activation, which are both triggered by oxidative stress,^11^ may favor a sympathetic activation.^12^ In any case proinflammatory substances and mediators may trigger signal to the central nervous system activating the sympathetic neurogenic component.^12^ The evidence on a direct role of inflammatory and immune factors in the development and/or in the progression of hypertension-related sympathetic activation is still lacking in human beings. Recently, however, some evidence confirming the importance of the inflammatory process in the neuroadrenergic activation has been provided in patients affected by rheumatoid arthritis, in which sympathetic nerve traffic recording displayed a level of neuroadrenergic activation greater for magnitude than the one detected in age-matched healthy controls.^13^ The SNS overdrive was also significantly potentiated when rheumatoid arthritis was detected in hypertensive patients.^13^

The second area of intense research in the field of the mechanisms of the sympathetic overdrive in hypertension refers to the contribution of genetic factors as potential triggers for the neurogenic alterations. In particular some specific genes have been identified. These include, for example, the phosphoducin gene, which is a regulator of G-protein-mediated signaling and is known to be present in several tissues, including the central nervous system and the sympathetic ganglia.^14^ Evidence has been provided that both in experimental animals and in humans this gene participates at the development of some forms of high blood pressure, such as those related to stress.^15^ In gene association studies evidence has been provided that subjects homozygous for the G allele of a phosphoducin single nucleotide polymorphism display greater blood pressure and sympathetic values than those found in humans with the A allele.^15^ Other studies have shown that subjects with a deficiency in genes encoding the melanocortin-3 and -4 receptors are characterized by low blood pressure and hemodynamic and biochemical signs of a reduced sympathetic tone.^16^ Conversely, genetic variations in neuropeptide Y receptors as well as in renalase activity appear to be associated with high blood pressure.^17^ Two final evidences further strengthen the role of genetic background in determining the sympathetic neural activation. Indeed in a study performed by our group we have shown that in hypertensive patients with metabolic syndrome, SNS overdrive is linked to the gene encoding the alpha_1A_ adrenoceptor.^18^ Similarly, Australian investigators have provided evidence that a single nucleotide polymorphisms in the norepinephrine transporter gene is linked to elevated circulating plasma levels of norepinephrine and elevated systolic (but not diastolic) blood pressure.^19^ Taken together, the findings suggest that some hypertensive clinical phenotypes may be characterized by a genetic background that is linked to the sympathetic neural abnormalities, thereby favoring the development with time of the elevated blood pressure values.

A further step forward in assessing the complex interrelationships between genetic factors and the SNS function comes from the studies which recently assessed microneurographic recording of efferent postganglionic sympathetic nerve traffic in patients with genetic hemochromatosis, the most common human model of primary iron overload.^20^ The unique feature of the study was represented by the sympathetic nerve traffic evaluation which was performed in these patients via the microneurographic technique before and after iron depletion therapy. Results provide strong evidence on the concept that iron overload is characterized by a hyperadrenergic state which is almost completely reversed by iron depletion. Recently, information on the role of iron, and specifically body iron overload, in the determination of the adrenergic overdrive typical of hypertensive state have been implemented, with the evidence that essential hypertensive patients with iron overload display sympathetic nerve traffic values significantly greater than the ones detected in uncomplicated hypertensives, for similar age and 24-hour blood pressure loads.^21^ This finding, strengthened by the significant relationships found in our study between muscle sympathetic nerve traffic, serum ferritin, transferrin saturation, and hepatic iron load, supports the hypothesis that iron “*per se*” is an organic metallic compound capable to exert sympathoexcitatory properties. Evidence has been indeed provided that iron is involved in a number biological steps that may directly or indirectly enhance sympathetic cardiovascular drive, including baroreflex alterations, vascular inflammation, immune reactivity, development of an oxidative stress process, and a nitric oxide crowing.

## THE SNS IN RESISTANT AND PSEUDORESISTANT HYPERTENSION

**Figure 2.**
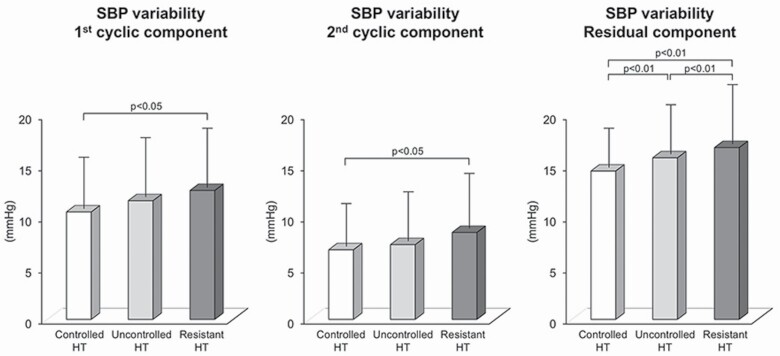
Behavior of the cyclic and residual components of systolic blood pressure (SBP) variability in controlled, uncontrolled, and resistant hypertensive (HT) patients of the Pressioni Arteriose Monitorate e Loro Associazioni study. Data are shown as the mean ± SEM. *P* < 0.05 and *P* < 0.01 refer to the statistically significant differences between groups.

In the past decade, growing interest has been focused on the different pathophysiological features of resistant hypertension, i.e., the clinical condition characterized by elevated blood pressure values despite the use of 3 or more antihypertensive agents, including a diuretic, even when employed at a full daily dosage.^22^ This is the case also for the involvement of SNS in the disease development and progression. An early study based on norepinephrine spillover assessment in 10 patients with resistant hypertension undergoing renal denervation has documented an increase in renal sympathetic drive, which has been more recently confirmed in a larger patients sample.^3,23^ More direct information on the patterns of the SNS in resistant hypertension come from data collected by our group in the Pressioni Arteriose Monitorate e Loro Associazioni (PAMELA) project, an epidemiological study carried out in a large sample of the general population living in the surroundings of the north Milan urban area.^24^ The aim of this specific substudy was to assess indirectly or directly the behavior of the adrenergic neural influences on the heart and peripheral circulation in resistant hypertensive patients.^25^ In the first set of studies, we examined the 2 cyclic components of systolic and diastolic blood pressure residual variability identified via a fast Fourier transform spectral analysis of the ambulatory blood pressure tracing. The first of these 2 components has been shown to be closely related to muscle sympathetic nerve traffic values, for both the systolic and the diastolic blood pressure components. When this analysis was applied to a selected group of hypertensive patients from the PAMELA study, we found that this first component was significantly greater in the case of systolic blood pressure in resistant hypertensives compared with the values seen in controlled and uncontrolled hypertensives, i.e., patients in which antihypertensive drug treatment was able or unable, respectively, to reduced blood pressure below 140/90 mm Hg (Figure 2). This was also the case for the second cyclic component and the residual variability values for systolic but not for diastolic blood pressure.

More direct evidence on the participation of neuroadrenergic factors in the development of a resistant hypertensive state comes from microneurographic data collected by our group in 2 different studies. The first study compared muscle sympathetic nerve traffic values recorded in a group of true resistant hypertensive patients to those found in 35 nonresistant hypertensives and in 19 healthy normotensive controls.^26^ Results show a marked potentiation (about 50%) of the adrenergic activation in resistant hypertensive patients as compared with age-matched nonresistant hypertensives and true normotensives. Of interest was the finding that spontaneous baroreflex sensitivity was significantly impaired in resistant hypertensives, which also displayed a significant relationship between muscle sympathetic nerve traffic values, anthropometric data, and plasma aldosterone levels.^26^ Similar findings were observed in 2 other studies performed in resistant hypertensive patients.^27,28^ In a more recent microneurographic study we further investigated the issue, by examining whether and to what extent true resistant hypertension displays a SNS profile different from the one seen in apparent or pseudoresistant hypertension, i.e., patients in which careful examination revealed that the poor blood pressure control was not dependent on a resistant blood pressure state but rather by a poor compliance and adherence to treatment.^29^ This latter clinical condition is remarkably different from the true resistant hypertensive state and depends on multiple and heterogeneous causes, including inadequate blood pressure measurements, nonadherence to treatment as well as presence of a white-coat effect. The microneurographic data collected by our group strongly support the notion that also the behavior of SNS is consistently different in these 2 clinical conditions. Indeed, at variance from the true resistant hypertensive state, apparent resistant hypertension does not display any sign of cardiac or peripheral sympathetic overactivation, the resulting neuroadrenergic profile being thus indistinguishable from the one characterizing the true normotensive state.^29^ In reviewing the data collected during the last decade on the topic related to the participation of SNS in development of resistant hypertension, it should be mentioned that special attention has been paid to the proadrenergic role of sleep apnea syndrome, which has been successfully documented in a number of studies.^30–32^

## HEART RATE A SYMPATHETIC BIOMARKER

In the past decade, attempts have been made to find simple but reliable markers of sympathetic cardiovascular function for being used in daily clinical practice. One of them might be represented by resting clinic heart rate for a number of reasons. First, heart rate has been shown to be an independent marker of cardiovascular risk in a variety of clinical conditions, including essential hypertension.^33^ Second, this variable has been documented in several studies to be elevated in hypertensive patients as compared with healthy controls, paralleling the degree of the neuroadrenergic overdrive.^33^ Third, a significant correlation between heart rate and cardiac norepinephrine spillover has been found in untreated hypertensive patients.^34^ Finally, in a number of disease characterized by a marked adrenergic overdrive, such as obesity, metabolic syndrome, heart failure, and hypertension, a significant correlation was detected between resting clinic heart rate and venous plasma norepinephrine or muscle sympathetic nerve traffic.^35^ Although some exceptions to this general rule may exist,^36^ there is overall an agreement on the possibility that assessment of clinic heart rate may provide useful insights on the functional status of the SNS in different clinical conditions.

**Figure 3.**
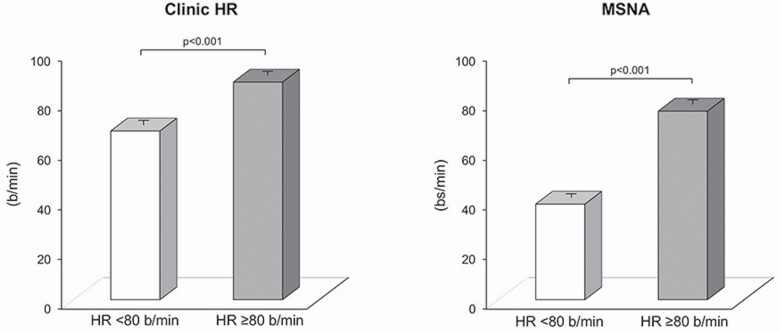
Clinic heart rate (HR, left panel) and muscle sympathetic nerve traffic (MSNA, right panel) in hypertensive patients subdivided in 2 groups according to resting clinic heart rate values below or above 80 beats/minute. Data are shown as the mean ± SEM. *P* < 0.01 refers to the statistically significant differences between groups. From data of ref. ^38^.

The clinical relevance of heart rate measurement has received further support from the inclusion of elevated heart rate in the list of the cardiovascular risk factors published in the latest European Society of Cardiology/European Society of Hypertension Guidelines on essential hypertension.^37^ The document identifies clinic heart rate greater than 80 beats/minute as the cutoff value above which cardiovascular risk is augmented.^37^ Of particular interest is the recent finding that heart rate values greater than 80 beats/minute closely mirror an increase in sympathetic cardiovascular drive, as assessed by clinical microneurography (Figure 3).^38^ Of note in the same study was the observation that a similar relationship was detected when 24-hour heart rate values or venous plasma norepinephrine levels were taken into account. Altogether these findings therefore emphasize the importance of assessing heart rate for obtaining information not only on the cardiovascular risk profile of a given hypertensive patient but also for determining, with a quite satisfactory approximation, the level of the existing sympathetic cardiac drive.

## LATEST FINDINGS FROM META-ANALYSES

**Figure 4.**
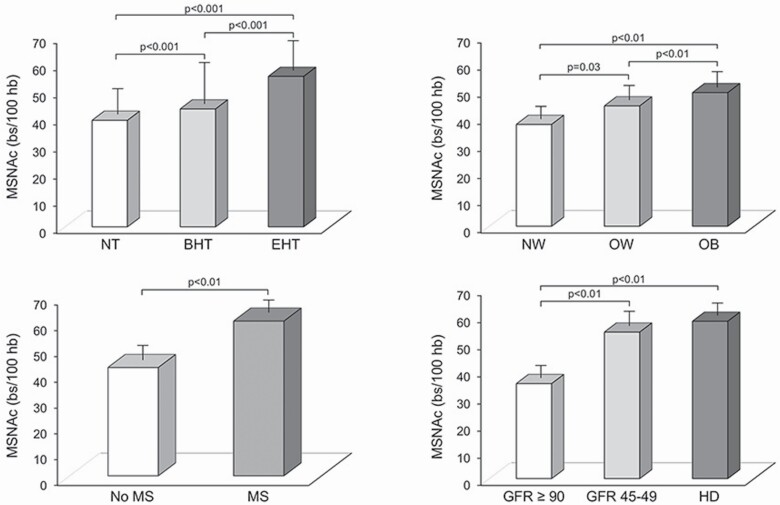
Behavior of muscle sympathetic nerve traffic (MSNA) in hypertension (upper left panel), obesity (upper right panel), metabolic syndrome (lower left panel), and chronic kidney disease (lower right panel). Data are shown as mean ± SD and refer to the results of 4 different meta-analysis. Abbreviations: BHT, borderline hypertensive patients; EHT, essential hypertensive patients; GFR, glomerular filtration rate; MS, metabolic syndrome; NT, normotensive subjects; NW, normoweight; O, obese subjects; OW, overweight. *P* < 0.01 and *P* < 0.001 refer to the statistically significant differences between groups. From data of refs. ^41–44^.

Meta-analytic studies recently contributed to improve the knowledge on the main features of the sympathetic activation in hypertension and related cardiovascular or metabolic disease, replacing the studies carried out more than 40 years ago examining the behavior of plasma norepinephrine in large cohorts of hypertensive or obese individuals.^39,40^ The unique feature of these studies was the assessment of the behavior of muscle sympathetic nerve traffic directly evaluated via the microneurographic technique in different groups of patients, i.e., essential hypertensives, obese subjects, patients with metabolic syndrome, and chronic renal failure.^41–44^ The results can be summarized as follows. First, in the 63 microneurographic studies on hypertension meta-analyzed for a total of 1,216 patients^41^ sympathetic nerve traffic was (i) significantly and progressively more elevated from the normotensive to the mild and the more sustained hypertensive state (Figure 4, left upper panel), (ii) related to clinic and to a greater extent to 24-hour ambulatory blood pressure values, and (iii) directly and significantly related to venous plasma norepinephrine and left ventricular mass index.^41^ Second, in the meta-analysis performed in 1,438 obese or overweight subjects recruited in 45 microneurographic studies,^42^ a progressive increase in sympathetic nerve traffic values was detectable from the normoweight to the overweight and the obese state (Figure 4, right upper panel), even when obstructive sleep apnea was excluded from the data analysis.^42^ At variance from the previously mentioned hypertensive population in obese subjects neither heart rate nor venous plasma norepinephrine values were significantly related to sympathetic nerve traffic. Similar results were obtained in the 16 microneurographic published studies recruiting more than 400 patients with metabolic syndrome (Figure 4, lower left panel).^43^ Recently, our group evaluated the behavior of sympathetic nerve traffic in patients with chronic kidney disease in a meta-analysis involving 638 patients with different chronic kidney disease severity recruited in 29 studies.^44^ The results (Figure 4, right lower panel) provide evidence on the fact that sympathetic activation in chronic kidney disease becomes more and more pronounced as the chronic kidney disease progresses toward kidney failure and closely relates not only to renal function but also to body mass index, age, and heart rate. Taken together the results of these meta-analytic studies have allowed to strengthen the results of single microneurographic studies, providing solid conclusions based on large population samples and thus overcoming one of the limitations of the published studies based on sympathetic nerve traffic recordings in humans, i.e., the limited sample size of the patients evaluated.

It should also be emphasized that one of the leading new concepts emerged in the last decade in the field of the assessment of the sympathetic neural function in cardiovascular disease refers to the evidence that the adrenergic overdrive reported in a number of cardiac, metabolic, and renal disease does not homogeneously affect the heart and the peripheral circulation and that a heterogeneous behavior may be detected in assessing adrenergic influences.^45^ This certainly makes more difficult and more complex the assessment of human sympathetic function in a variety of pathologic conditions.

## SNS RESPONSES TO RENAL DENERVATION AND CAROTID BARORECEPTOR STIMULATION

The last decade has seen a remarkable interest for considering sympathetic activation at the center of the pathophysiology of essential hypertension and thus as a potential target of the therapeutic intervention. Two procedures in particular have raised the interest of investigators and clinicians, i.e., carotid baroreceptor stimulation and bilateral renal nerves ablation.^3^ Carotid baroreceptor stimulation has been shown to exert marked blood pressure lowering effects which are mediated by the sympathoinhibitory properties of the procedure capable to substantially reduce (average reduction 30%–40%) muscle sympathetic nerve traffic.^46^ Of special interest is the observation that the procedure when employed in congestive heart failure, i.e., a clinical condition characterized by a massive sympathetic activation,^47^ may trigger profound sympathoinhibitory effects with a significant improvement in the clinical severity of the disease, as assessed by the New York Heart Association (NYHA) functional class.^48^

Reflecting the controversy generated by the analysis of the heterogeneous blood pressure effects of renal denervation, the results of the procedure on sympathetic nerve traffic, and more in general on sympathetic cardiovascular drive, were also far from being univocal. As mentioned in a previous section of this review, while renal norepinephrine spillover undergoes after renal denervation a substantial reduction,^3^ microneurographic investigations produced conflicting results, some studies showing a sympathoinhibitory effect while other no change.^49–52^ In our own experience, one of the potential problems related to the evaluation of the effects of bilateral renal nerves ablation on SNS refers to the fact that the neurogenic and the blood pressure effects of the procedure do not necessarily share the same temporal pattern of response.^53^ Indeed in a series of 15 patients with resistant hypertension undergone bilateral renal denervation we examined whether and to what extent the blood pressure responses to the procedure display a pattern qualitatively and quantitatively similar to the one of SNS. To clarify this issue, a serial measurement of clinic and ambulatory blood pressure values, together with sympathetic nerve traffic recording, was performed before and after 2 weeks, 4 weeks, and 6 months from the procedure. Results provide evidence that the sympathoinhibition and the blood pressure lowering effects do not share the same temporal response, characterized by an early reduction in blood pressure and by a late decrease in sympathetic nerve traffic. Taken together these findings suggest that the 2 phenomena are not necessarily linked by a cause–effect relationship and that the blood pressure lowering responses to the procedure in the earlier phase after renal denervation might be mediated by mechanisms other than the SNS ones.

## RESIDUAL RISK AND SYMPATHOINHIBITION

Many studies have shown that despite the favorable effects of antihypertensive drug treatment on cardiovascular morbidity and mortality the cardiovascular risk of treated hypertensive patients remains higher than the one seen in the control normotensive population, even when blood pressure values are reduced to or almost to the recommended blood pressure targets.^54,55^ Several are the possible factors which may be involved in the phenomenon. These include the poor control of associated cardiovascular risk factors, as shown in many intervention studies, due also to the fact the multifactorial risk profile makes control of the individual risk components much more difficult.^37^ It may also depend on the genetic profile which makes cardiovascular risk unmodifiable with intervention on blood pressure values. A further mechanism relates to the late initiation of antihypertensive drug treatment, when functional or structural cardiovascular alterations associated with a high blood pressure state become scarcely modifiable by treatment. An additional mechanism, closely related to the sympathetic abnormalities reported in essential hypertension, should be mentioned. Namely the fact that antihypertensive drug treatment, despite its ability to exert sympathoinhibitory effects, does not appear to completely reverse the adrenergic overdrive related to hypertension, leaving the sympathetic function higher than the one seen in the true normotensive individuals.^5^ This may mean that a not negligible portion of the SNS activation is irreversible and that this phenomenon may adversely affect the cardiovascular risk profile of the treated hypertensive patient.

## CONCLUSIONS AND PERSPECTIVES

Data reviewed in this paper document the continuous interest of clinicians and investigators for defining the sympathetic abnormalities characterizing the hypertensive state together with their impact on cardiovascular risk profile, target organ damage, and clinical outcome. Directions for future research in the area include an in-depth analysis of (i) the pathophysiological link between genetic background and the neurogenic abnormalities characterizing hypertension, (ii) the crosstalks between the immune system and the sympathetic neural function, and (iii) the potential impact of antihypertensive combination drug treatment on the neuroadrenergic function, with a specific analysis of what this may mean for the impact of the so-called “residual sympathetic activation” on the residual cardiovascular risk.
